# Planned Subtotal Resection following Stereotactic Radiosurgery of Koos 3 and 4 Vestibular Schwannomas

**DOI:** 10.3390/jcm13144107

**Published:** 2024-07-14

**Authors:** Grzegorz Turek, Sebastian Dzierzęcki, Paweł Obierzyński, Adrian Drożdż, Zenon Mariak, Justyna Zielińska-Turek, Wojciech Czyżewski, Karolina Dżaman, Mirosław Ząbek

**Affiliations:** 1 Department of Neurosurgery, Bródnowski Masovian Hospital, 03-242 Warsaw, Poland; 2 Gamma Knife Centre, 03-242 Warsaw, Poland; 3 Department of Descriptive and Clinical Anatomy, Center of Biostructure Research, Medical University of Warsaw, 02-004 Warsaw, Poland; 4 Department of Neurosurgery, Medical University of Białystok, 15-276 Białystok, Poland; zenon.mariak@umb.edu.pl; 5 Department of Neurology, National Medical Institute of the Ministry of the Interior and Administration, 02-507 Warsaw, Poland; 6 Department of Neurosurgery, Maria Skłodowska-Curie National Research Institute of Oncology, 02-781 Warsaw, Poland; wojciech.w.czyzewski@gmail.com; 7 Department of Didactics and Medical Simulation, Medical University of Lublin, 20-954 Lublin, Poland; 8 Department of Otolaryngology, Centre of Postgraduate Medical Education, 03-242 Warsaw, Poland; 9 Department of Neurosurgery, Centre of Postgraduate Medical Education, 03-242 Warsaw, Poland

**Keywords:** large vestibular schwannoma, gamma knife surgery, microsurgery, subtotal resection, facial nerve preservation

## Abstract

**Background/Objectives**: Surgical resection of medium to large vestibular schwannomas (VSs, Koos grade 3 and 4) is a widely used approach, although stereotactic radiosurgery (SRS) is increasingly proposed as initial treatment. The quality of life-centered approach is challenged in cases where tumor growth control cannot be achieved with SRS, thus necessitating salvage surgery. We present a series of eight consecutive patients who required surgery due to continued tumor growth after SRS. **Methods**: Of the 146 patients with VS grades 3 and 4 initially treated with SRS, only eight patients (mean age, 54 ± 7.2 years; range, 42–63 years) required subsequent surgery. Their mean tumor volume was 9.9 ± 3.2 cm^3^. The mean time from SRS to first tumor progression and planned subtotal resection was 23 ± 5.9 months and 45 ± 17.5 months, respectively. SRS was not performed after the surgery in favor of a “wait and rescan” approach. Tumor residue was monitored on follow-up magnetic resonance imaging. In all patients, tumor growth control after planned subtotal resection was maintained at 63 ± 19.8 months. **Results:** None of the 146 patients had serious complications after SRS. In the eight patients who required surgery, tumor growth between 22% and 212% (mean, 4 cm^3^) was reported within 26 to 84 months after SRS. Before salvage surgery, they scored 1 point on the House–Brackmann scale. Subtotal excision was performed, and VIIth nerve function was preserved in all patients. At 63 ± 19.8 months, 3 patients had a House–Brackmann score of 1, four patients had a score of 2, and one patient had a score of 3. **Conclusions:** Surgical excision of medium to large VS after SRS can be relatively safe, provided that a quality of life-centered approach of subtotal resection is used.

## 1. Introduction

Vestibular schwannomas (VSs) account for approximately 6% to 8% of all intracranial tumors [1,2]. At the time of diagnosis, even up to 45% of VSs touch or compress the brainstem and are therefore classified as “larger” tumors (grade 3 and 4 according to the Koos scale) [3,4,5]. Surgical resection of VSs is usually the treatment of choice. It is often recommended for medium to large tumors based on favorable outcomes of large clinical series reported by highly specialized centers [6,7,8,9,10,11,12,13]. While still the gold standard, this invasive approach has recently been challenged by the quality of life-centered approach using radiosurgery as initial treatment.

The near-perfect results described by leading centers are difficult to replicate: many authors report good facial function in only 27% to 58% of patients after gross total resection, even with state-of-the-art microsurgical and neuromonitoring techniques [6,7,8,9,10,11,12,13]. Today, when patients expect complete resolution of symptoms, such low rates of functional success can be disappointing and inconsistent with the popular notion of what modern surgery can achieve. At the same time, stereotactic radiosurgery (SRS) has been consistently shown to provide high tumor growth control (up to 87%) and low complication rates [13,14,15,16], encouraging its use as the treatment of choice even for larger VSs. However, in medium to large VSs adjacent to major neurovascular structures (thus limiting available space for surgical manipulation), determining eligibility for SRS vs microsurgery is challenging, even in highly experienced centers with access to both techniques. One of the potential concerns is the high level of technical difficulty along with an increased risk of mortality and morbidity if tumor growth control cannot be achieved with SRS and surgery is required.

The challenges associated with tumor excision following SRS have been addressed by several clinical reports, but evidence regarding larger VSs is scarce. The few case reports available in the literature have mainly focused on the total vs. subtotal resection approach with respect to the risk of subsequent tumor regrowth [17,18]. However, the question of which treatment modality is optimal in these cases remains largely unanswered. 

To address this gap in knowledge, we present findings based on data from eight of the 146 patients with medium to large VSs initially treated with SRS who required subsequent surgery due to insufficient control of tumor size. In all cases, subtotal partial excision was deliberatively planned and performed, resulting in preservation of VIIth nerve function and no tumor growth during a mean follow-up of 63 months. 

## 2. Materials and Methods

Between March 2011 and March 2021, 1587 patients with VSs were referred for SRS by a tumor board at our tertiary care neurosurgical center. All patients were informed about the available treatment modalities (i.e., microsurgery and radiosurgery) as well as about the potential sequelae of radiosurgery, including inadequate tumor volume control, hearing loss, facial nerve dysfunction, and exacerbation of symptoms due to brainstem compression. In 146 of the 1587 patients, VSs were classified as medium to large (grades 3 and 4 according to Koss scale, where grade 1 indicates small intracanalicular tumor; grade 2, small tumor with protrusion into the cerebellopontine cistern; grade 3, medium-size tumor occupying the cerebellopontine cistern without brainstem displacement; and grade 4, large tumor with brainstem displacement). Of the 146 patients, eight did not respond to radiosurgery and underwent salvage resection of the tumor to reduce clinical symptoms associated with further tumor growth and/or cerebral edema.

The study was conducted in accordance with the ethical standards of the relevant committee on human experimentation and with the Declaration of Helsinki, and approved by the Ethics Committee of the Centre of Postgraduate Medical Education in Warsaw, Poland (protocol code 155/2022; approval date, 2022).

### 2.1. Gamma Knife Radiosurgery

The main goal of radiosurgery was to control tumor growth and to preserve facial nerve function as well as hearing (if still serviceable). All procedures were performed with a Leksell Gamma Knife Perfexion unit with 192 sources of cobalt-60 (Elekta, Stockholm, Sweden). A conformal plan with 4-mm isocenters was constructed using Leksell Gamma Plan (Elekta). One-millimeter 3D T1-weighted, FSE T2-weighted, and FIESTA-weighted magnetic resonance images were used for planning. Highly conformal planning was considered particularly important along the anterior margin of the tumor as well as at the fundus of the internal auditory canal, close to the cochlea. In all eight patients, the prescribed tumor dose was 12 Gy to the 50% isodose line. Special attention was paid to the cochlear dosimetry, with close monitoring of both the maximum and mean doses. The mean cochlear dose was 7.5 Gy, ranging from 5.6 to 9.9 Gy.

All 146 patients with medium to large VSs underwent a routine follow-up visit at 12 months after GKS and then every 12 months thereafter. The follow-up examination was performed by the operating surgeon (GT) in cooperation with a radiologist. Facial nerve function was assessed using the House–Brackmann scoring system, and pure tone audiometry and speech discrimination test results were expressed using the Gardner–Robertson scale. Tumor volume was assessed by the manual delineation of tumor borders on axial contrast-enhanced T1-weighted magnetic resonance images, introduced in the Elekta Gamma Plan 10 follow-up module. A favorable radiosurgical outcome was defined as a reduction or stabilization of tumor size. Any increase in tumor volume greater than 20% of the volume measured on the day of irradiation was considered inadequate control of tumor growth and thus an eligibility criterion for salvage surgery. Patients who showed tumor growth were followed closely, and the decision on salvage treatment was made only after transient swelling (pseudoprogression) was excluded (unless patients developed symptoms of brainstem compression). Among all 146 patients transient swelling (pseudoprogression) was reported in 9%.

### 2.2. Salvage Surgery

Tumor excision was performed using a retrosigmoid approach with mandatory facial nerve monitoring. In all cases, the challenge for the surgeon was not so much the identification of the nerve, but rather its dissection from the firm adhesions present in each patient. Since any serious damage to the VIIth nerve was considered unacceptable, an approach of intentional subtotal tumor resection was adopted in all patients. Thus, any manipulation within the internal auditory canal was avoided, because the “lip” of this structure was shown to be the most vulnerable site for VIIth nerve damage. Therefore, a thin layer of the tumor capsule adjacent to the facial nerve was always left intact to ensure that the anatomical and functional continuity of the nerve is preserved. This was achieved in all patients. 

Another problem during the surgery was to deal with significant adhesions of the tumor mass to the brainstem, which were present in all eight patients. However, in contrast to the dissection of the VIIth nerve, after careful preparation, the tumor could be separated from the brainstem without any noticeable worsening of the signs and symptoms that could indicate brainstem damage. 

In each patient, the postoperative clinical status was assessed at discharge and then at 6 and 12 months. During follow-up, patients were evaluated for facial and cochlear nerve function and the presence of new signs and symptoms. Tumor volume was estimated for the first time at 6 months after surgery and then every 12 months.

## 3. Results

### 3.1. Basic Characteristic of the Study Group

**Figure 1:**
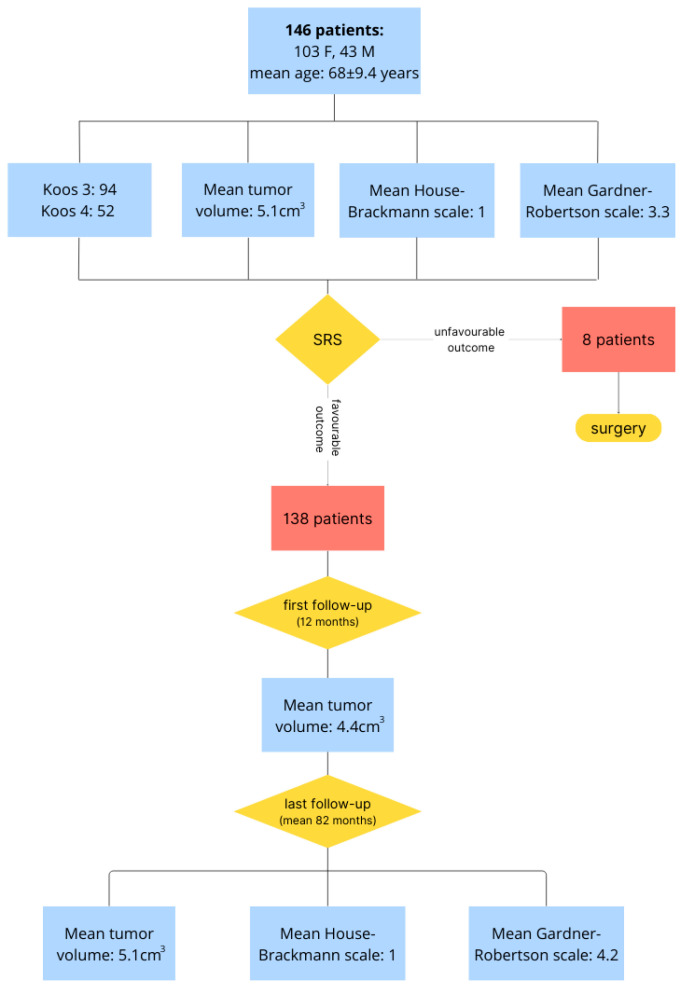
Characteristics and outcome of 146 patients with Koos 3 and 4 vestibular schwannomas treated with primary SRS. F, female; M, male; SRS, stereotactic radiosurgery.

The characteristics and outcomes of 146 patients with Koos 3 and 4 VSs are presented in Figure 1. The clinical status and outcomes of tumor imaging in the eight patients (five women and three men) who underwent salvage surgery are presented in Table 1 and Table 2. The mean age of these eight patients was 54 ± 7.2 years at diagnosis. Before gamma knife surgery (GKS), all patients experienced progressive hearing impairment and tinnitus. Vestibular syndrome was reported in five patients, while pain/dysesthesia over the distribution of the Vth nerve was reported in four patients. The mean tumor volume before GKS was 5.9 ± 2.5 cm^3^ (3.2–10.2 cm^3^). Koos grade 3 VS was reported in two patients, and grade 4 in six patients. The mean time between radiosurgery and first radiological signs of tumor progression was 23 ± 5.9 months (range, 12–32 months). The mean time between radiosurgery and salvage microsurgery was 45 ± 17.5 months (range, 26–84 months). 

### 3.2. Outcome of Gamma Knife Radiosurgery

**Figure 2:**
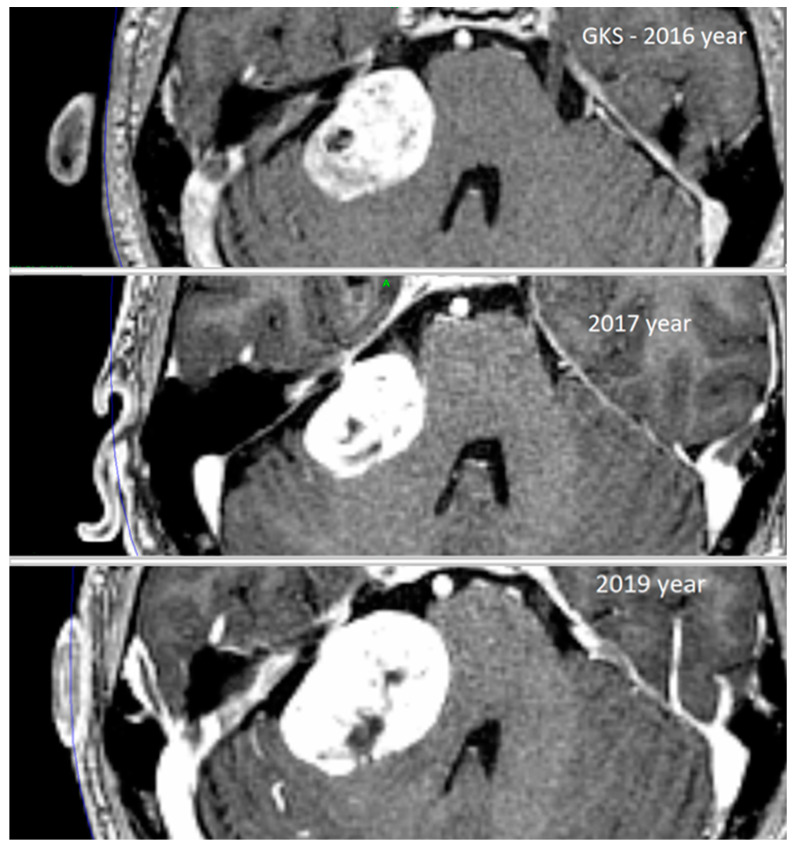
Patient No. 1. Enhanced magnetic resonance image showing the progression of right-sided giant vestibular schwannoma at the time of gamma knife surgery in 2016, a reduced tumor volume at the first follow-up visit in 2017 year, and progression of tumor volume at the last follow-up visit before surgical resection in 2019.

Tumor progression was noted in eight of the 146 patients undergoing GKS, and these patients were further assessed in this case series. Tumor volume increased by a minimum of 22% to a maximum of 212% within 26 to 84 months after GKS. The mean increase in tumor volume was 4.0 ± 2.7 cm^3^: from 5.9 ± 2.5 cm^3^ to 9.9 ± 3.2m^3^, *p* = 0.015 (Figure 2). In three patients, volume progression was noted in the solid part of the tumor, and in one patient in the cystic compartment. In the remaining four patients, tumor volume increased due to the expansion of the central necrotic part of the tumor (Table 2). Additionally, in three patients, edema of the neighboring brainstem and/or cerebellum was reported at 42, 50, and 84 months after SRS (Table 2).

GKS did not cause any serious impairment of brainstem and/or cerebellar function or any lasting functional damage to the facial nerve in any of the patients. Specifically, no worsening of any of the previous signs and symptoms from these cardinal neural structures neighboring the tumor mass was observed. Almost all patients scored 1 point on the House–Brackmann scale before and immediately after GKS (Table 1). 

The function of nerve VIII was more sensitive to GKS than that of nerve VII. Hearing deteriorated in five of the eight patients (mean 3.6 ± 0.7 after GKS vs. 2.9 ± 1.6 at baseline, based on the Gardner–Robertson grading scale) (Table 1). Intermediate audition between SRS treatment and salvage surgery revealed deterioration of hearing to non-serviceable already at first visit with tumor volume progression in three patients who presented serviceable hearing before SRS. However, patients with tinnitus or vertigo before GKS did not show a worsening of symptoms. In addition, none of the patients developed tinnitus or vertigo as a de novo symptom after GKS. Of the five patients with sensory deficits within the Vth nerve distribution, one patient developed trigeminal neuralgia at 36 months after GKS. Another patient developed ataxia and gait disturbance at 24 months. However, as these new symptoms occurred with a significant delay after SRS, they can be considered a consequence of tumor expansion rather than a direct result of radiosurgery. 

### 3.3. Outcome of Surgery

As mentioned above, all patients scored 1 on the House–Brackmann scale at the time of tumor resection. At discharge, two patients maintained a score of 1, while three patients scored 2 and another three patients scored 3. At the last follow-up visit after a mean of 63 months, VIIth nerve function improved in three patients: in one patient, the score improved from 2 to 1, and in two patients from 3 to 2. In the remaining five patients, VIIth nerve function remained stable: two patients maintained a score of 1 point, two patients a score of 2 points, and one patient a score of 3 points on the House–Brackmann scale. 

At the time of salvage surgery, all patients presented with non-serviceable hearing levels based on the Gardner–Robertson scale: three patients showed grade 3 (non-serviceable), four patients grade 4 (poor hearing), and one patient grade 5 (none/deaf) (Table 1). At the last follow-up visit, further deterioration of hearing was observed: six patients had grade 4 and two patients grade 5.

All patients demonstrated transitional nystagmus when recovering from anesthesia, which gradually resolved within days or weeks after surgery. No lower cranial nerve (IX-XII) deficits were noted either before or after surgery.

**Figure 3:**
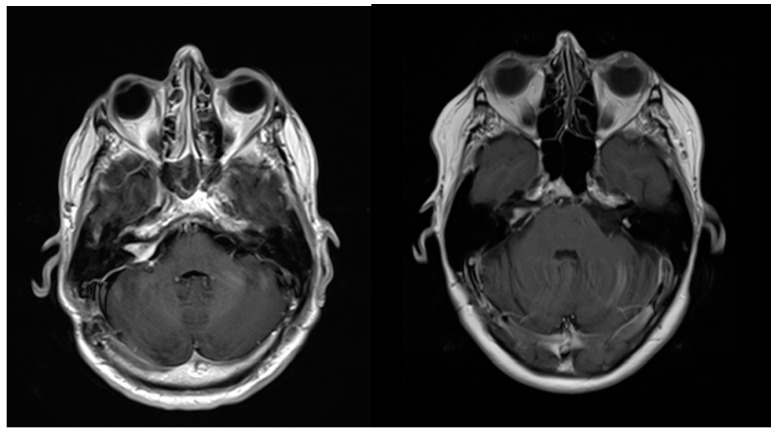
Patient No. 1. Enhanced magnetic resonance image showing right-sided vestibular schwannoma 6 months after planned subtotal resection. No increase in tumor volume was observed at 57 months of follow-up (right-sided image).

Magnetic resonance imaging was routinely performed at 6 months after surgery. A significant reduction in tumor volume from 9.9 ± 3.2 cm^3^ to 1.5 ± 0.6 cm^3^ was observed in all patients, *p* < 0.001 (Table 2, Figure 3). In addition, a further reduction in tumor residuals was observed at 63 months to a mean of 0.9 ± 0.6 cm^3^ in all patients (*p* = 0.142, Table 2). 

Histopathological examination revealed a typical benign pattern of VSs in all patients. No malignant transformation was observed during follow-up.

## 4. Discussion

### 4.1. Initial Gamma Knife Surgery

After years of controversy, SRS has gradually emerged as a viable option for the initial treatment of VSs [8,16,19,20,21,22,23]. Different clinical series reported tumor control (with preservation of VIIth nerve function) in 81% to 98% of patients [8,16,17,19,20,21,22,23]. In addition, a recent meta-analysis showed that this approach is effective in patients with VSs grades 3 and 4 [23]. However, these optimistic findings must be interpreted with caution, especially when dealing with larger tumors. It was consistently shown that tumor volume is the only independent negative predictor of successful SRS outcome [13,14,15,23,24]. An SRS response rate of 94.1% was reported for tumors smaller than 0.5 cm^3^ and only of 80.7% for tumors larger than 6 cm^3^. Moreover, tumor progression can occur even up to 3 years after an SRS session, while an increase in tumor volume (pseudoprogression) is relatively common during the first year after surgery. Therefore, the decision to perform salvage surgery must be carefully considered, especially when the option of repeated low-dose GKS is also acceptable and feasible: Yomo et al. presented very good outcomes of repeated SRS, but mainly for smaller tumors (Koos 1-2) [22]. According to Mindermann et al. [25], a decision on surgery should be considered at least 18 months after GKS, although Nonaka et al. [26] recommend extending this period up to even 3 years. In our patients, the decision on salvage surgery was made 45 ± 17.5 months after GKS, although tumor growth was detected at a mean of 25 months after radiosurgery, except in one patient where it was detected at 12 months. 

In our study, of the 146 patients with medium to large VSs (Koos 3-4) who underwent GKS, only eight patients required salvage surgery, resulting in a tumor growth control rate of 94.5%. Lee et al. [27] reported the rate of 98% for tumors of different sizes, with surgery performed in 13 of 600 patients (in whom tumor volume increased by 21% to 743% within 3 to 107 months after GKS). Importantly, in our study, none of the patients experienced worsening of symptoms of brainstem or cerebellum compression or impairment of facial nerve function after GKS. All our patients scored 1 on the House–Brackmann scale before and after radiosurgery. Therefore, our results for medium to large VSs are consistent with those reported for tumors of different sizes. Aboukais et al. [17] and Huang et al. [8] reported no complications, while in a case series of 37 patients by Wise et al. [28], three patients developed hydrocephalus and one patient developed delayed facial palsy 4 months after surgery. 

In our study, the VIIIth nerve appeared to be more sensitive to radiosurgery, and hearing deteriorated in all our patients after SRS. This is in line with the results of Wise et al. [28]: of 17 patients with serviceable hearing, only one patient maintained this level of hearing, while 15 patients showed complete hearing loss within 40 months after SRS.

### 4.2. Outcome of Microsurgery after SRS

Despite the high success rate reported for SRS, the question remains as to which approach is optimal in the relatively small population of patients who do not respond to initial radiosurgery. Clinicians are reluctant to use radiosurgery as a first-line treatment option for fear of future challenges if secondary surgery for larger VSs is required. At the same time, there is a need to address the long-standing problem of how radical the surgery should be to effectively control the tumor while preserving nerve function.

To answer these questions, we performed a retrospective analysis of the results obtained in eight of the 146 patients with medium to large VSs who required salvage surgery due to lack of tumor growth control after initial radiosurgery. Planned subtotal tumor resection using the retrosigmoid approach was performed in all eight patients, with satisfactory outcomes. The use of this approach was guided by the previous experience of world leaders in VS surgery, who acknowledged the lack of consensus to support the superiority of any surgical strategy with respect to the extent of VS resection and concluded that the main goal of the management of large VSs should focus on maintaining quality of life and making every effort to preserve facial or cochlear function [3].

Our case series revealed promising long-term results. All patients with preserved VIIth nerve function after radiotherapy generally also showed preserved function after salvage surgery. Patients scored from 1 to 3 on the House–Brackmann score after surgery, which can be considered a satisfactory outcome. As for VIIIth nerve function, serviceable hearing before radiosurgery was reported only in three of the eight patients but was not retained after radiosurgery. Thus, all patients presented with no serviceable hearing at salvage surgery. As expected, hearing also did not return to serviceable levels after surgery. Importantly, no deficit of the cranial nerves (IX-XII) was observed in any of the patients, which is in line with the results of Aboukais et al. [17] in patients with a wide range of tumor sizes who underwent surgery after SRS. 

Based on our findings, several pertinent questions can be addressed more directly. First, regarding concerns about technical difficulties with resection of a large tumor after previous SRS, we did indeed observe significant adhesions between the tumor and facial nerve fascicles in all our patients. Although Roche et al. [29] and Aboukais et al. [22] reported technical difficulties in only 43.5% and 60% of patients undergoing surgery after previous SRS, respectively, other authors reported such difficulties in almost all cases. Radiation-induced hypervascularization and fibrosis, changes in tumor shape and consistency, loss of the peritumoral arachnoid plane and/or arachnoid thickening were common and were identified as risk factors for surgical failure. In contrast, Shuto et al. [30] reported no consistent association between radiosurgery and increased adhesions between the tumor and the facial nerve.

Given the inherent technical difficulties, a cautious approach to salvage surgery is necessary when dealing with large VSs. In particular, such a surgery should not be guided by the ambition to completely remove the tumor. We used a preplanned partial tumor excision approach following Zhang et al. [31], Starnoni et al. [3], Gurgel et al. [21], Schwartz et al. [32], Wise et al. [28] and Iwai et al. [33] who clearly demonstrated that subtotal or even near-total resection reduces the rate of facial nerve injury. Due to the paucity of literature data on salvage excision of large VSs, it is difficult to make any definitive recommendation. However, good functional outcome in our patients and lack of tumor regrowth during 63-month follow-up seem to support our cautious approach of partial or near-total resection. 

Importantly, close neuromonitoring is required at all times to ensure safety, even when the goal is partial or near-total resection rather than complete removal. Recently, it has been suggested that to achieve better outcomes, simple mapping of the VIIth nerve should be replaced by functional monitoring to enable prognostic estimation of nerve survival. Specifically, in the case of large VSs, the monitoring of motor evoked potentials was shown to be useful in identifying the proximal facial nerve at advanced stages of tumor excision. As for cochlear nerve monitoring, although it is much more difficult to achieve, it should be used even when removing large tumors, provided that nerve function is preserved at the time of surgery. Neuromonitoring of the VIIIth nerve was not used in our patients because none of them had preserved hearing at the time of surgery. 

Another important issue, although somewhat loosely related to the major discussion, is the further management of patients in whom tumor fragments had not been resected, so as not to compromise the quality of life. So far, none of our patients has been scheduled for further SRS, although this cautious approach is based on our previous experience with radiosurgical treatment of VSs rather than on literature data. We only identified a single report that would address this issue [34]. Nevertheless, the lack of evidence of tumor regrowth in our patients at 63 months seems to support our approach with regular follow-up and no SRS as long as it is warranted (i.e., continued control of tumor growth confirmed by regular monitoring). 

### 4.3. Limitations

A limitation of this study is the relatively small number of patients who required salvage surgery after SRS. On the other hand, this limitation is due to the high efficacy of SRS in controlling Koos 3 and 4 VSs. On the basis of our previous experience, hardly any conclusion could be drawn as to if, how and when to perform SRS in the patients after salvage surgery. 

## 5. Conclusions

Our results indicate that surgical excision of large VSs after previous GKS, although commonly considered to be challenging and risky, can be still performed relatively safely, provided that a cautious approach of planned subtotal resection is used, with a focus on quality of life. Our approach seems to be effective: all eight patients who underwent salvage surgery showed stable tumor volume during 63 months of follow-up, thus indicating satisfactory tumor growth control. Further studies with longer follow-up are needed to provide more evidence and more definitive conclusions to guide the management of patients with tumor regrowth after initial SRS.

## Figures and Tables

**Table 1:** Characteristics of the eight patients referred for salvage surgery: clinical status.

Patient No.	Sex/ Age at Diagnosis	Symptoms at Diagnosis					House–Brackmann Scale				Gardner–Robertson Scale		
		Hearing Impairment	Tinnitus	Vestibular	Trigeminal	Ataxia	Before GKS	Before Surgery	Early after Surgery	At Last Follow-Up	Before GKS	Before Surgery	At Last Follow-Up
1.	M/53	Yes	Yes	Yes	Yes	Yes	1	1	2	1	1	3	4
2.	F/63	Yes	Yes	Yes	No	No	1	1	3	2	3	5	5
3.	F/51	Yes	Yes	No	No	No	1	1	3	3	2	3	4
4.	F/62	Yes	Yes	Yes	Yes	Yes	1	1	3	2	3	4	4
5.	M/42	Yes	Yes	No	Yes	No	1	1	1	1	1	3	4
6.	F/48	Yes	Yes	Yes	No	No	1	1	2	2	3	3	4
7.	F/59	Yes	Yes	No	Yes	Yes	1	1	2	2	5	4	4
8.	M/54	Yes	Yes	Yes	Yes	Yes	1	1	1	1	5	4	5
No of ‘Yes’		8	8	5	5		-	-	-	-	-	-	-

F, female; M, male; GKS, gamma knife surgery.

**Table 2:** Characteristics of the eight patients referred for salvage surgery: tumor imaging.

Patient No.	Tumor Size (Koos Scale)		Tumor Volume (cm^3^)				Dose (Gy)		Perifocal Edema after GKS	Type of Progression	Time From	
	Before GKS	Before Surgery	Before GKS	On Surgery	At 6 Months after Surgery	At Last Follow-Up	on Tumor	on Cochlea			GKS to Volume Progression (Months):	GKS to Surgery (Months):
1.	4	4	10.2	15.0	1.9	1.7	12	8.1	+	solid	32	42
2.	3	4	3.2	4.4	1.2	0.4	12	6.8	-	solid	12	26
3.	4	4	4.7	12.3	1.1	0.8	12	9.9	-	necrosis	24	36
4.	4	4	4.5	8.3	0.3	0.03	12	5.6	+	cystic	24	84
5.	4	4	7.7	9.4	2.2	1.9	12	7.6	-	necrosis	24	36
6.	4	4	8.2	11.7	1.6	1.2	12	8.5	+	necrosis	24	38
7.	3	4	3.9	7.7	1.2	0.5	12	6.2	-	solid	18	52
8.	4	4	5.0	10.3	2.1	1.1	12	7.5	-	necrosis	27	46
Mean:	3.75	4	5.9 ± 2.5	9.9 ± 3.2	1.5 ± 0.6	0.9 ± 0.6	12	7.5 ± 1.4	-	-	22 ± 5.9	45 ± 17.5

GKS, gamma knife surgery.

## Data Availability

The raw data supporting the conclusions of this article will be made available by the authors on request.
